# Association between oxidative balance score and skeletal muscle mass and strength: NHANES from 2011 to 2018

**DOI:** 10.3389/fnut.2024.1414161

**Published:** 2024-06-26

**Authors:** Xiaoxuan Zhao, Lijiangshan Hua, Kaili Jin, Qiuhua Sun, Rongyun Wang

**Affiliations:** Scholl of Nursing, Zhejiang Chinese Medical University, Hangzhou, China

**Keywords:** oxidative balance score, diet, lifestyle, skeletal muscle mass, handgrip strength

## Abstract

**Objective:**

Oxidative stress is a risk factor for sarcopenia. The Oxidative Balance Score (OBS) is a widely employed tool for evaluating the oxidative stress-related exposures from dietary and lifestyle factors. In this study, we aimed to conducted to explore the relationship between OBS and skeletal muscle mass and strength.

**Methods:**

6,438 subjects from 2011 to 2018 and 5,414 from 2011 to 2014 from the National Health and Nutrition Examination Survey (NHANES) were selected for analysis. The correlations between OBS and skeletal muscle mass and handgrip strength were investigated using multivariate logistic regression and linear regression analysis.

**Results:**

Compared with lowest OBS, participants with OBS in the highest quartile had lower risk of low skeletal muscle mass (OR = 0.173 (0.120 ~ 0.248), *p* < 0.0001) and low handgrip strength (*β* = 0.173 (0.120 ~ 0.248), *p* = 0.011). The negative association also were found between dietary/lifestyle OBS and skeletal muscle mass (OR = 0.268 (0.178 ~ 0.404), *p* < 0.0001; OR = 0.231 (0.130 ~ 0.410), *p* < 0.0001) and handgrip strength (*β* = 1.812 (0.555 ~ 3.071), *p* = 0.008; *β* = −2.255 (−3.430 ~ −1.079), *p* < 0.001) independently. The positive association remains significant, especially among men and those with higher education levels by subgroup analysis.

**Conclusion:**

All of these results indicated a negative association between OBS and low skeletal muscle mass and handgrip strength. An antioxidant-rich diet and healthy lifestyle are crucial for enhancing skeletal muscle mass and strength.

## 1 Introduction

Sarcopenia is defined as a progressive and generalized skeletal muscle disorder characterized by an accelerated decline in muscle mass and function (1). It is a common geriatric syndrome among the elderly (2). The prevalence ranges from 8 to 36% in individuals younger than 60 years of age and from 10 to 27% in people older than 60 years of age (3). Sarcopenia is associated with an increase in adverse outcomes including falls, functional decline, frailty, and mortality, severely impairing quality of life and mortality (4, 5). Given the rising occurrence of this long-lasting, advancing, and impairing illness and the growing challenge of treating it, it is crucial to discover novel and efficient approaches to prevent sarcopenia.

Oxidative stress is a critical factor in the process of muscle atrophy. Under conditions of aging and disease, the antioxidant capacity of skeletal muscle decreases, leading to a decrease in the cell’s ability to maintain the balance of the redox state, which results in oxidative damage (6). Excessive intracellular oxidative stress in skeletal muscle leads to a significant rise in the generation of reactive oxygen species (ROS). Excessive production of reactive oxygen species (ROS) in these processes can activate redox pathways in muscle fibers, resulting in oxidative damage, mitochondrial dysfunction, and inhibition of protein synthesis (7, 8). These factors affect muscle regenerative capacity and contribute to both muscle loss and decreased strength (9). Hence, skeletal muscle benefits from low levels of reactive oxygen species (ROS), whereas an excessive ROS concentration can impede its function. Therefore, it is essential to establish effective protective mechanisms against oxidation, especially in skeletal muscles highly susceptible to this process. Antioxidants seem to be a rational approach to address the decline in muscle mass and function (10).

The Oxidative Balance Score (OBS) represents a comprehensive assessment of the overall oxidative balance. It is derived from evaluating both the pro-oxidant and antioxidant components present in Individual’s diet and lifestyle. A higher OBS indicates a higher level of antioxidant exposure compared to pro-oxidant exposure (11). OBS has been employed in numerous epidemiological studies to assess the risk of various chronic diseases linked to oxidative stress, including chronic kidney disease (12), depression (13), diabetes (14) and periodontitis (15), etc. However, the relationship between OBS and sarcopenia-related traits, especially the decrease in muscle mass and strength, remains unknown. Therefore, this study aims to investigate the potential association between them.

## 2 Methods

### 2.1 Study population

**Figure 1:**
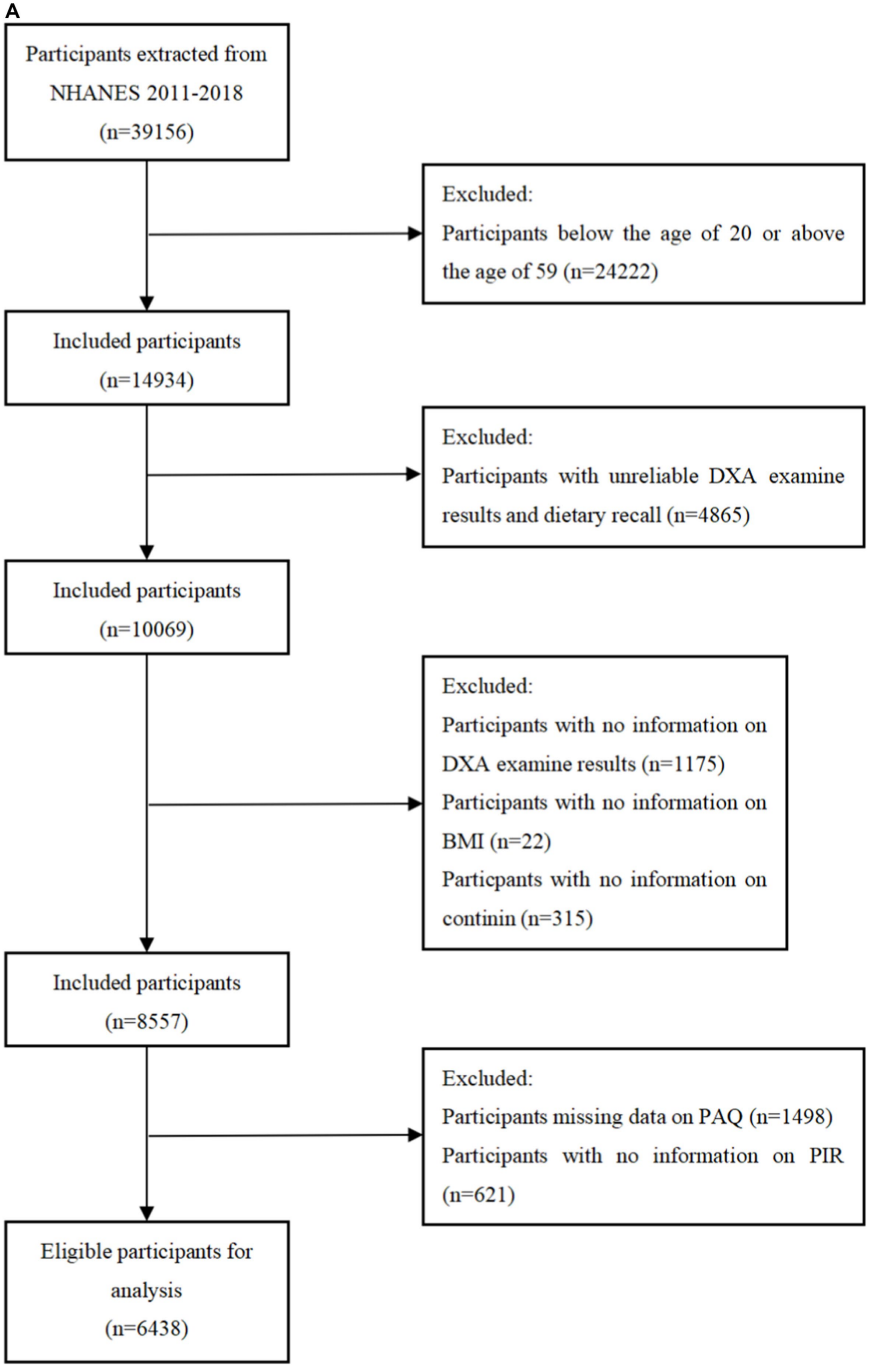

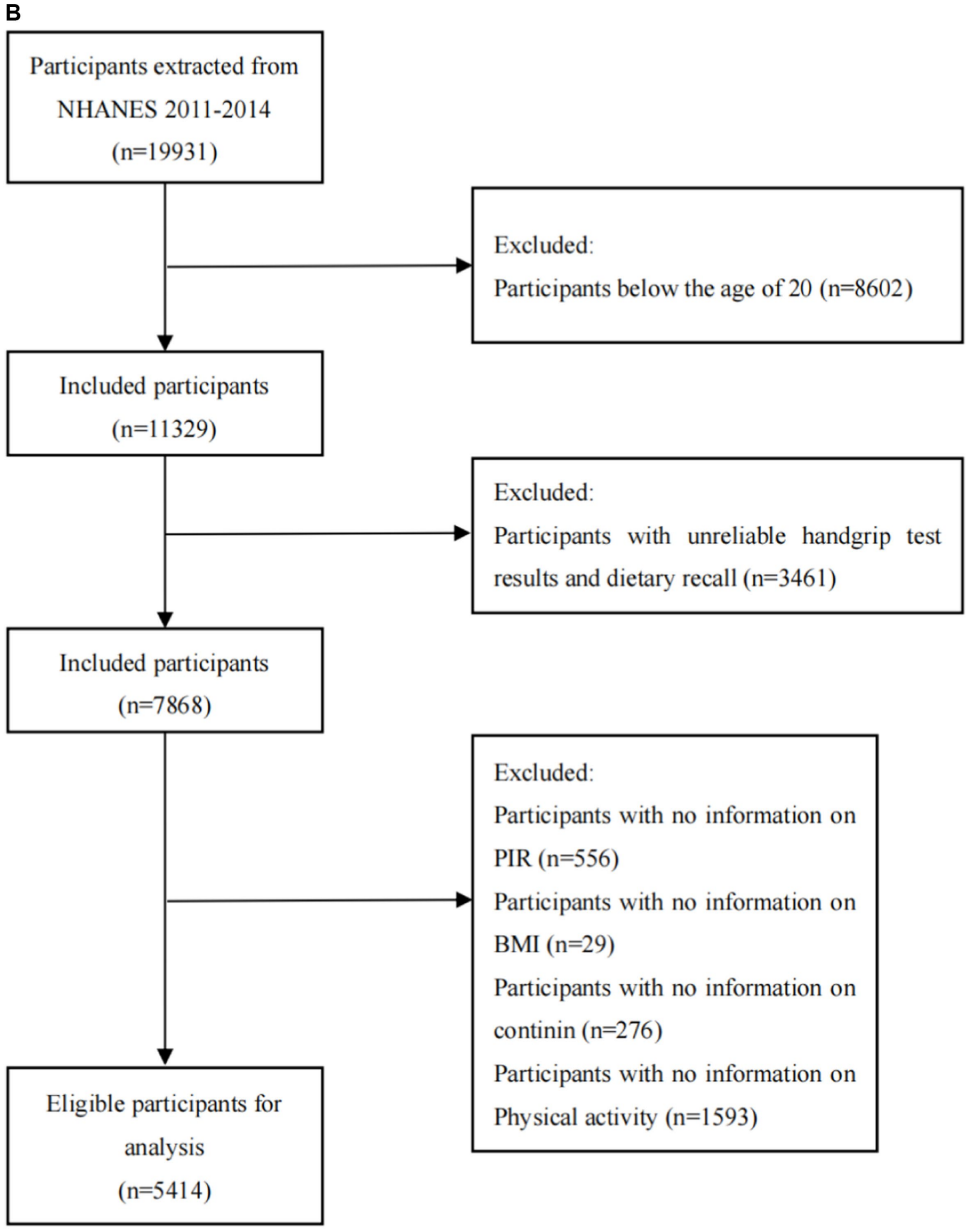
**(A)** Flowchart of the sample selection from NHANES 2011–2018. **(B)** Flowchart of the sample selection from NHANES 2011–2014.

The National Health and Nutrition Examination Survey (NHANES) is a research program aimed to assess the nutritional status and general health of adults and children in the United States. This study program focuses on various population groups or health issues and combines physical tests and questionnaires. The prevalence of important diseases and risk factors for disease will be ascertained using the survey’s data. Data will be analyzed to evaluate nutritional status and how it relates to disease prevention and health promotion. All surveys were approved by the Ethical Review Board (IRB) of the National Center for Health Statistics and written consent was obtained from the participants. Among 39156 participants from the NHANES 2011 to 2018, participants were excluded if they were under 20 years old, had unreliable data (*n* = 4,865), or lacked information on DXA measurements (*n* = 1,175), BMI (*n* = 22), cotinine (*n* = 315), PAQ (*n* = 1,498), PIR (*n* = 621). From the 19931 participants between 2011 and 2014, participants who had unreliable grip strength test results or two 24-h dietary recalls (*n* = 3,461), lacked data of BMI (*n* = 29), PIR (*n* = 556), cotinine (*n* = 276), and physical activity (*n* = 1,593) were excluded. Finally, a total of 6,438 participants from the NHANES 2011 to 2018 and 5,414 participants form the NHANES 2011 to 2014 were included in this research (Figures 1A,B).

### 2.2 Oxidative balance scores

The OBS was calculated by totalizing 16 nutrients and 4 lifestyle factors, which consisted of 15 antioxidants and 5 pro-oxidants (16, 17). Table 1 described the OBS scheme. Our nutrient and alcohol intake data were obtained from two 24-h dietary recall interviews conducted by NHANES in partnership with the United States Department of Agriculture (USDA) and the United States Department of Health and Human Services (DHHS). The National Center for Health Statistics (NCHS) Health and Nutrition Examination Surveys Division of the DHHS oversaw all aspects of the survey sample design and data collection. The Food Surveys Research Group (FSRG) of the USDA was responsible for the dietary data collection methodology, maintenance of the database used for coding and processing the data, as well as data review and processing. For alcohol intake, we allocated 2 points to non-drinkers, 1 point to non-heavy drinkers (0–15 g/d for women and 0–30 g/d for men), and 0 points to heavy drinkers (≥15 g/d for women and ≥ 30 g/d for men) (17). Physical activity data were gathered from the Physical Activity Questionnaire (PAQ) in NHANES, administered by trained interviewers using a computer-assisted personal interviewing (CAPI) system. The calculation of physical activity included work-related activities (high and moderate intensity) as well as leisure-time activities (such as commuting on foot or bicycle, high-intensity leisure activities, and moderate-intensity leisure activities). Following established methodology, physical activity was quantified as metabolic equivalent (MET) scores multiplied by the weekly frequency and duration of each type of activity (18). We utilized serum cotinine concentration as an indicator to evaluate smoking status, which is a major proximal metabolite of nicotine and can be employed to indicate active smoking as well as exposure to secondhand smoke. We grouped all components except alcohol intake by gender and then divided them into three groups based on their tertiles. Antioxidants were assigned 0 to 2 points in ascending order from the lowest to the highest tertile, while pro-oxidants were assigned in reverse, with 0 points in the highest tertile and 2 points in the lowest tertile. Subsequently, we calculated the total points to include in the analysis (17).

**Table 1:** Ingredients that make up the oxidative balance score.

OBS components	Population	Property	Male			Female		
			0	1	2	0	1	2
**Dietary OBS components**								
Dietary fiber (g/d)	a	A	<27.10	27.10–44.10	≥44.10	<22.90	22.90–35.90	≥35.90
	b		<27.70	27.70–44.60	≥44.60	<23.90	23.90–36.50	≥36.50
Carotene (RE/d)	a	A	<1163.00	1163.00–3784.00	≥3784.00	<1275.00	1275.00–4288.00	≥4288.00
	b		<1250.74	1250.74–4043.37	≥4043.37	<1336.46	1336.46–4647.54	≥4647.54
Riboflavin (mg/d)	a	A	<3.53	3.53–5.25	≥5.25	<2.76	2.76–3.95	≥3.95
	b		<3.50	3.50–5.17	≥5.17	<2.78	2.78–3.98	≥3.98
Niacin (mg/d)	a	A	<49.70	49.70–69.50	≥69.50	<34.10	34.10–48.20	≥48.20
	b		<47.02	47.02–67.15	≥67.15	<33.71	33.71–47.18	≥47.18
Vitamin B6 (mg/d)	a	A	<3.68	3.68–5.47	≥5.47	<2.64	2.64–3.92	≥3.92
	b		<3.63	3.63–5.42	≥5.42	<2.70	2.70–3.97	≥3.97
Total folate (mcg/d)	a	A	<681.00	681.00–1035.00	≥1035.00	<520.00	520.00–785.00	≥785.00
	b		<680.74	680.74–1028.00	≥1028.00	<532.46	532.46–800.00	≥800.00
Vitamin B12 (mcg/d)	a	A	<7.21	7.21–12.55	≥12.55	<4.97	4.97–8.67	≥8.67
	b		<7.25	7.25–12.41	≥12.41	<5.15	5.15–8.82	≥8.82
Vitamin C (mg/d)	a	A	<78.60	78.60–198.70	≥198.70	<76.40	76.40–175.90	≥175.90
	b		<88.97	88.97–209.26	≥209.26	<84.14	84.14–188.46	≥188.46
Vitamin E (ATE) (mg/d)	a	A	<13.40	13.40–21.20	≥21.20	<11.40	11.40–17.60	≥17.60
	b		<13.12	13.12–20.93	≥20.93	<11.23	11.23–17.42	≥17.42
Calcium (mg/d)	a	A	<1596.00	1596.00–2421.00	≥2421.00	<1264.00	1264.00–1912.00	≥1912.00
	b		<1535.00	1535.00–2365.79	≥2365.79	<1275.46	1275.46–1893.08	≥1893.08
Magnesium (mg/d)	a	A	<540.00	540.00–769.00	≥769.00	<432.00	432.00–595.00	≥595.00
	b		<531.00	531.00–754.26	≥754.26	<437.00	437.00–601.00	≥601.00
Zinc (mg/d)	a	A	<20.20	20.20–29.40	≥29.40	<14.50	14.50–20.80	≥20.80
	b		<19.74	19.74–28.70	≥28.70	<14.71	14.71–20.57	≥20.57
Copper (mg/d)	a	A	<2.09	2.09–3.03	≥3.03	<1.70	1.70–2.43	≥2.43
	b		<2.11	2.11–3.02	≥3.02	<1.75	1.75–2.46	≥2.46
Selenium (mcg/d)	a	A	<221.00	221.00–310.00	≥310.00	<158.00	158.00–224.00	≥224.00
	b		<212.07	212.07–297.70	≥297.70	<154.40	154.40–219.90	≥219.90
Total fat (g/d)	a	P	≥212.00	147.00–212.00	<147.00	≥158.00	110.00–158.00	<110.00
	b		≥204.35	138.68–204.35	<138.68	≥152.69	106.21–152.69	<106.21
Iron (mg/d)	a	P	≥36.80	25.50–36.80	<25.50	≥27.70	19.20–27.70	<19.20
	b		≥36.74	25.85–36.74	<25.85	≥28.36	19.64–28.36	<19.64
**Lifestyle OBS components**								
Physical activity (MET-minute/week)	a	A	1800.00	1800.00–6480.00	≥6480.00	<1100.00	1100.00–3480.00	≥3480.00
	b		1440.00	1440.00–4800.00	≥4800.00	<920.00	920.00–2760.00	≥2760.00
Alcohol (g/d)	a	P	≥30.00	0.00–30.00	None	≥15.00	0.00–15.00	None
	b		≥30.00	0.00–30.00	None	≥15.00	0.00–15.00	None
Body mass index (kg/m^2^)	a	P	≥29.90	25.40–29.90	<25.40	≥31.40	24.90–31.40	<24.90
	b		≥29.70	25.40–29.70	<25.40	≥31.10	24.90–31.10	<24.90
Cotinine (ng/mL)	a	P	≥5.40	0.02–5.40	<0.02	≥0.10	0.01–0.10	<0.01
	b		≥0.61	0.02–0.61	<0.02	≥0.06	0.01–0.06	<0.01

A, antioxidant; P, pro-oxidant; RE, retinal equivalent; ATE, alpha-tocopherol equivalent; MET, metabolic equivalent.a, participants from NHANES 2011–2018. b, participants from NHANES 2011–2014.

### 2.3 Assessment of skeletal muscle mass and hand grip strength

Skeletal muscle mass was measured by Dual-energy X-ray absorptiometry (DXA), which is characterized by low radiation levels, precise differentiation between various tissue components, and the capacity for repeated measurements. This measurement involves determining the total lean body mass of the arms and legs and can be conducted efficiently within a short time. The measurement excludes individuals who are pregnant, have self-reported the use of radiographic contrast (such as barium) within the past 7 days, weigh more than 450 pounds, or are taller than 6 feet and 5 inches. Prior to the examination, it is standard procedure to remove all metal objects, with the exception of dentures and hearing aids. Low skeletal muscle mass was defined according to the criteria established by the Foundation for the National Institutes of Health (FNIH). It is characterized by a ratio of low skeletal muscle mass adjusted for body mass index (ASM/BMI) that is less than 0.512 for females and less than 0.789 for males (19).

Muscle strength is assessed using the Combined Grip Strength from the Hand Grip Strength Test. Participants squeezed the dynamometer as hard as possible with one hand while standing and tested each hand three times, alternating hands between tests and resting the same hand for 60 s between measurements. Participants who were unable to take the test with both hands and who were unable to stand were excluded. Combined grip strength (kg) is the sum of the maximum grip strength values for each hand.

### 2.4 Covariates

Covariates age, gender (female, male), race (Mexican American, non-Hispanic white, non-Hispanic black, others), education (below high school, high school or above), family poverty-income ratio (PIR) (poor, not poor), Hypertension, diabetes mellitus and hyperlipidemia based on the previous studies (13, 20, 21). Hypertension is determined by medication use. Based on medical professionals’ self-reported diagnoses, diabetes mellitus and hyperlipidemia were identified.

### 2.5 Statistical analysis

All our data were weighted according to the NHANES analysis guidelines, considering the complex sampling design of NHANES. Data were analyzed using the R (4.2.2) software package, and statistical significance was determined at a two-sided *p*-value of 0.05. The baseline characteristics were categorized into quartiles based on the total OBS score. Each variable was represented as a weighted percentage and was regarded as categorical. The median is used to represent continuous variables. Differences between groups were assessed using the chi-square test and Kruskalallis rank sum test. We categorized the total OBS into quartiles, setting Q1 as the control group. Subsequently, we constructed three weighted multifactorial logistic regression models to evaluate the association between OBS and skeletal muscle mass and strength. We selected covariates with positive significance (*p* < 0.05) to adjust weighted multifactor logistic regression models. The result was showed in Supplementary Table A1. We also included the individual medians of the OBS total score quartiles in the trend analysis and computed trend *p*-values (22), and validated using restricted cubic spline plots (RCS). The association between OBS and skeletal muscle mass and strength in individuals of age, gender, education and PIR was examined using subgroup analysis. A sensitivity analysis was performed by systematically eliminating each factor from the adjusted model 3.

## 3 Results

### 3.1 Baseline characteristics

In this study, 11852 subjects were recruited, 6,438 for the relationship between OBS and skeletal muscle mass, and 5,414 for the relationship between OBS and hand grip strength. The participants’ baseline characteristics, as indicated by the OBS quartile, are detailed in Tables 2, 3. Compared to the lowest OBS quartile, a higher proportion of individuals in the high OBS quartile were non-Hispanic white and married or partnered. Furthermore, those in the high OBS quartile tended to have higher levels of educational attainment and family income. Additionally, there was a decreasing percentage of hypertensive and diabetic individuals as the OBS increased. There were no statistically significant differences between groups in terms of age, gender, or prevalence of hyperlipidemia (*p* > 0.05).

**Table 2:** The baseline characteristics by quartiles of the OBS: NHANES 2011–2018.

Characteristic	All	Q1 (<15)	Q2 (15–20)	Q3 (20–26)	Q4 (≥26)	*p* value
	*N* = 6,438	*N* = 1,533	*N* = 1,419	*N* = 1,727	*N* = 1,759	
Age (%)						0.108
Below30	1735 (27.0)	442 (30.9)	367 (26.5)	441 (25.6)	485 (27.9)	
30–40	1616 (24.0)	354 (20.8)	357 (24.9)	449 (24.6)	456 (27.4)	
40–50	1601 (24.8)	390 (24.9)	351 (20.8)	444 (24.8)	416 (20.5)	
Above50	1486 (24.2)	347 (23.4)	344 (27.8)	393 (24.9)	402 (24.2)	
Gender (%)						0.086
Male	3272 (50.4)	730 (50.4)	767 (51.7)	869 (50.5)	906 (49.2)	
Female	3166 (50.0)	803 (49.6)	652 (48.3)	858 (49.5)	853 (50.8)	
Race (%)						<0.001
Non-Hispanic White	2487 (64.4)	562 (62.1)	520 (63.3)	699 (66.2)	706 (65.3)	
Non-Hispanic Black	1321 (11.0)	419 (13.6)	332 (15.1)	292 (9.3)	278 (7.4)	
Other	1752 (15.5)	370 (15.2)	379 (13.5)	498 (16.3)	505 (16.3)	
Mexican American	878 (9.1)	182 (9.1)	188 (8.0)	238 (8.2)	270 (10.9)	
Marital (%)						0.019
Yes	3867 (64.0)	856 (58.1)	853 (63.4)	1076 (66.8)	1082 (66.3)	
No	2571 (36.0)	677 (41.9)	566 (36.6)	651 (33.2)	677 (33.7)	
Education (%)						<0.001
Below high school	924 (11.2)	267 (14.1)	226 (11.9)	219 (9.7)	212 (9.7)	
High school or above	5514 (88.8)	1266 (85.9)	1193 (88.1)	1508 (90.3)	1547 (90.3)	
PIR (%)						0.003
Not poor	5123 (85.1)	1152 (81.3)	1132 (85.0)	1418 (87.7)	1421 (85.7)	
Poor	1315 (14.9)	381 (18.7)	287 (15.0)	309 (12.3)	338 (14.3)	
Diabetes (%)						0.001
Yes	434 (5.0)	140 (7.2)	105 (5.5)	114 (3.9)	75 (4.1)	
No	6004 (95.0)	1393 (92.8)	1314 (94.5)	1613 (96.1)	1684 (95.9)	
Hypertension (%)						0.005
Yes	1097 (16.3)	316 (19.3)	249 (15.6)	272 (14.8)	260 (15.9)	
No	5341 (83.7)	1217 (80.7)	1170 (84.4)	1455 (85.2)	1499 (84.1)	
Hyperlipidemia (%)						0.243
Yes	733 (22.5)	181 (22.6)	169 (26.6)	189 (20.7)	194 (21.3)	
No	5705 (77.5)	1352 (77.4)	1250 (73.4)	1538 (79.3)	1565 (78.7)	
ASM/BMI (%)						<0.001
Yes	471 (5.7)	157(10.9)	106(6.3)	134(6.6)	74(2.1)	
No	5967 (94.30)	1376(89.1)	1313(93.7)	1593(93.4)	1685(97.9)	

**Table 3:** The baseline characteristics by quartiles of the OBS: NHANES 2011–2014.

Characteristic	All	Q1 (<13)	Q2 (13–19)	Q3 (19–24)	Q4 (≥24)	*p* value
	*N* = 5,414	*N* = 1218	*N* = 1416	*N* = 1199	*N* = 1581	
Age (%)						0.112
Below40	2150 (39.1)	455 (39.9)	553 (40.1)	458 (36.6)	684 (45.2)	
40–60	1883 (38.7)	407 (38.9)	465 (35.7)	441 (40.2)	570 (35.7)	
60–70	806 (13.5)	214 (13.6)	218 (14.5)	173 (13.3)	201 (11.8)	
70–80	575 (8.7)	142 (7.6)	180 (9.8)	127 (9.9)	126 (7.2)	
Gender (%)						0.24
Male	2790 (51.0)	617 (50.1)	768 (55.1)	596 (49.5)	809 (52.1)	
Female	2624 (49.0)	601 (49.9)	648 (44.9)	603 (50.5)	772 (47.9)	
Race (%)						<0.001
Mexican American	583 (7.5)	110 (8.1)	147 (7.8)	131 (7.8)	195 (8.5)	
Non-Hispanic Black	1165 (9.8)	370 (15.8)	341 (12.2)	217 (8.2)	237 (6.2)	
Non-Hispanic White	2396 (70.5)	498 (63.9)	602 (66.6)	558 (72.2)	738 (71.9)	
Other	1270 (12.2)	240 (12.2)	326 (13.4)	293 (11.8)	411 (13.4)	
Marital (%)						0.03
No	2200 (36.2)	545 (43.4)	589 (38.5)	463 (35.3)	603 (34.9)	
Yes	3214 (63.8)	673 (56.6)	827 (61.5)	736 (64.7)	978 (65.1)	
Education (%)						<0.001
Below high school	861 (11.4)	258 (15.9)	261 (13.2)	158 (9.4)	184 (8.4)	
High school or above	4553 (88.6)	960 (84.1)	1155 (86.8)	1041 (90.6)	1397 (91.6)	
PIR (%)						0.003
Not poor	4331 (86.2)	909 (80.8)	1136 (85.5)	982 (87.9)	1304 (88.1)	
Poor	1083 (13.8)	309 (19.2)	280 (14.5)	217 (12.1)	277 (11.9)	
Diabetes (%)						0.013
No	4789 (91.3)	1040 (88.8)	1238 (92.6)	1056 (91.5)	1455 (94.0)	
Yes	625 (8.7)	178 (11.2)	178 (7.4)	143 (8.5)	126 (6.0)	
Hypertension (%)						0.018
No	4010 (76.8)	851 (73.2)	1019 (75.4)	895 (76.1)	1245 (82.1)	
Yes	1404 (23.2)	367 (26.8)	397 (24.6)	304 (23.9)	336 (17.9)	
Hyperlipidemia (%)						0.563
No	4566 (83.4)	1017 (81.8)	1193 (83.0)	1002 (84.8)	1354 (84.3)	
Yes	848 (16.6)	201 (18.2)	223 (17.0)	197 (15.2)	227 (15.7)	
Grip (median [IQR])		71.13 [56.30, 92.24]	73.30 [57.42, 91.86]	69.50 [56.30, 92.10]	73.47 [58.00, 95.60]	0.251

### 3.2 Association between OBS and skeletal muscle mass and strength

As demonstrated in Table 4, among participants from 2011 to 2018, we examined the association between OBS and low skeletal muscle mass using a weighted logistic regression model. Among participants from 2011 to 2014, weighted linear regression models were used to analysis the relationship between OBS and handgrip strength. All models consistently showed that OBS was negatively associated with the risk of low skeletal muscle mass and strength in Model 3, after adjusting for all relevant factors, the highest quartile of OBS showed a stronger negative association with the risk of low skeletal muscle mass (OR = 0.173 (0.120, 0.248), *p* < 0.001) and handgrip strength (*β* = 1.499 (0.392, 2.606), *p* = 0.011) compared to the lowest quartile of OBS. It was indicated that the probability of low skeletal muscle mass decreased by 82.7% for each unit increase in OBS when OBS was above 24. Compared to the lowest OBS quartile, the second and third OBS quartiles exhibited a significant negative association with the risk of low skeletal muscle mass, and all of these correlations were found to be statistically significant (Q2: OR = 0.526 (0.349, 0.792), *p =* 0.003; Q3 OR = 0.571 (0.397, 0.821), *p* = 0.003). There was significant trend by the trend test except in model 1 (*p* for trend <0.0001). In sensitivity analysis, the same trend was observed (Supplementary Table A2).

**Table 4:** Weighted logistic and linear regression analysis models for the associations between OBS and skeletal muscle mass and strength.

OBS	Model 1	*p* value	Model 2	*p* value	Model 3	*p* value
**Skeletal muscle mass(OR, 95%CI)**						
Q1	Ref		Ref		Ref	
Q2	0.551 (0.371,0.819)	0.004	0.503 (0.334, 0.758)	0.001	0.526 (0.349, 0.792)	0.003
Q3	0.580 (0.407,0.826)	0.003	0.541 (0.375, 0.781)	0.001	0.571 (0.397,0.821)	0.003
Q4	0.178 (0.125,0.253)	<0.0001	0.161 (0.113, 0.229)	<0.0001	0.173 (0.120,0.248)	<0.0001
*p* for trend		<0.0001		<0.0001		<0.0001
**Handgrip strength (β, 95% CI)**						
Q1	Ref		Ref		Ref	
Q2	1.014 (−1.139,3.166)	0.344	−0.372 (−1.479,0.735)	0.490	−0.371 (−1.497,0.754)	0.494
Q3	0.293 (−1.604,2.189)	0.755	0.833 (−0.328,1.993)	0.150	0.884 (−0.303,2.071)	0.134
Q4	3.033 (0.656,5.410)	0.014	1.490 (0.398,2.582)	0.010	1.499 (0.392,2.606)	0.011
*p* for trend		0.057		<0.001		<0.001

Model 1: Unadjusted model. Model 2: Skeletal muscle mass: Adjusted for age, race, education and poverty-income ratio. Handgrip strength: Adjusted for gender, age, race, education, marital and poverty-income ratio. Model 3: Additionally adjusted for hypertension, diabetes and hyperlipemia. The specific range for the quantiles is consistent with Tables 2, 3.

### 3.3 Association between the dietary OBS/lifestyle OBS and skeletal muscle mass and strength

We conducted a separate analysis to assess the association between dietary and lifestyle OBS and sarcopenia using multivariate logistic regression. The results of this analysis are presented in Table 5. In adjusted models, both high dietary OBS and lifestyle OBS showed a significant association with a low risk of skeletal muscle mass (dietary OBS: OR = 0.268 (0.178, 0.404), *p* < 0.0001, lifestyle OBS: OR = 0.224 (0.126, 0.396), *p* < 0.0001) and handgrip strength (dietary OBS: *β* = 1.812 (0.555, 3.071), *p* < 0.0001, lifestyle OBS: *β* = −2.255 (−3.430, −1.079), *p* < 0.0001). The trend was statistically significant in all model 2 and model 3 (*p* < 0.0001).

**Table 5:** Weighted logistic and linear regression analysis models showing the associations between dietary/lifestyle OBS and skeletal muscle mass and strength.

OBS	Q1	Q2	*p* value	Q3	*p* value	Q4	*p* value	*p* for trend
**Skeletal muscle mass (OR, 95%CI)**								
**Dietary OBS**								
Model 1	Ref	0.777 (0.571, 1.057)	0.106	0.635 (0.434,0.929)	0.020	0.303 (0.202, 0.455)	<0.0001	<0.0001
Model 2	Ref	0.677 (0.489, 0.937)	0.020	0.569 (0.380, 0.853)	0.007	0.257 (0.171, 0.385)	<0.0001	<0.0001
Model 3	Ref	0.694 (0.501, 0.961)	0.028	0.588 (0.395, 0.873)	0.010	0.268 (0.178, 0.404)	<0.0001	<0.0001
Lifestyle OBS								
Model 1	Ref	0.898 (0.589, 1.369)	0.610	0.580 (0.404, 0.832)	0.004	0.194 (0.114, 0.333)	<0.0001	<0.0001
Model 2	Ref	0.850 (0.555, 1.301)	0.446	0.613 (0.424, 0.886)	0.010	0.219 (0.124, 0.386)	<0.0001	<0.0001
Model 3	Ref	0.881 (0.560, 1.385)	0.575	0.627 (0.428, 0.919)	0.018	0.231 (0.130, 0.410)	<0.0001	<0.0001
**Handgrip strength (β, 95%CI)**								
**Dietary OBS**								
Model 1	Ref	0.606 (−1.538, 2.749)	0.568	0.302 (−1.724, 2.328)	0.762	2.988 (0.414, 5.562)	0.024	0.021
Model 2	Ref	−0.461 (−1.583, 0.662)	0.401	0.954 (−0.244, 2.152)	0.112	1.817 (0.582, 3.052)	0.006	<0.001
Model 3	Ref	−0.485 (−1.633, 0.662)	0.383	0.982 (−0.239, 2.205)	0.108	1.812 (0.555, 3.071)	0.008	<0.001
**Lifestyle OBS**								
Model 1	Ref	−0.242 (−2.877, 2.392)	0.852	−1.123 (−3.093, 0.846)	0.252	2.592 (−0.259, 5.444)	0.073	0.052
Model 2	Ref	−0.987 (−2.152, 0.179)	0.093	−1.598 (−2.966, −0.230)	0.024	−2.223 (−3.367, −1.091)	<0.001	<0.001
Model 3	Ref	−1.015 (−2.187, 0.158)	0.085	−1.636 (−3.001, −0.271)	0.022	−2.255 (−3.430, −1.079)	<0.001	<0.001

Model 1: Unadjusted model. Model 2: Skeletal muscle mass: Adjusted for age, race, education and poverty-income ratio. Handgrip strength: Adjusted for gender, age, race, education, marital and poverty-income ratio. Model 3: Additionally adjusted for hypertension, diabetes and hyperlipemia. The specific range for the quantiles: Skeletal muscle mass: Dietary OBS: Q1 [1,10); Q2 [10,16); Q3 [16,22); Q4 [22,31]; Lifestyle OBS: Q1 [1,3); Q2 [3,4); Q3 [4,6); Q4 [6,8]. Handgrip strength: Dietary OBS: Q1 [2,10); Q2 [10,16); Q3 [16,22); Q4 [22,31]; Lifestyle OBS: Q1 [0,3); Q2 [3,4); Q3 [4,5); Q4 [5,7].

### 3.4 Subgroup analyses

We conducted subgroup analyses based on the characteristics of different populations to clarify the relationship between OBS and skeletal muscle mass and strength across varied demographic groups. In the group with a high school group or above, compared with participants with the lowest OBS, subjects in Q4 had a lower risk of lower appendicular skeletal muscle (OR = 0.138 (0.086, 0.221), *p* < 0.0001, *p* for interaction = 0.019). Among the men, higher OBS plays a more significant role in handgrip strength (*β* = 3.806 (1.237, 6.375), *p* = 0.006, *p* for interaction = 0.009). The negative association between OBS and skeletal muscle mass and strength was substantially stronger in individuals with a higher PIR as compared to those with a low PIR. Interestingly, results from subgroup analyses by age showed that the higher the OBS, the lower the risk of low muscle mass and the higher handgrip strength, compared to Q1, in those under 60 years of age (Table 6).

**Table 6:** Subgroup analysis for associations between OBS and appendicular skeletal muscle mass and strength.

Subgroup	Q1	Q2	*p* value	Q3	*p* value	Q4	*p* value	*p* for interaction
**Skeletal muscle mass (OR, 95%CI)**								
Education								0.019
Below high school	Ref	1.073 (0.476, 2.417)	0.863	0.952 (0.379, 2.393)	0.915	0.480 (0.218, 1.054)	0.067	
High school or above	Ref	0.452 (0.274, 0.746)	0.003	0.509 (0.331, 0.783)	0.003	0.135 (0.083, 0.220)	<0.0001	
PIR								0.057
Not poor	Ref	0.577 (0.360, 0.925)	0.023	0.630 (0.412, 0.964)	0.034	0.145 (0.087, 0.242)	<0.0001	
Poor	Ref	0.338 (0.148, 0.775)	0.011	0.312 (0.141, 0.692)	0.005	0.310 (0.163, 0.588)	0.001	
Age								0.104
<30	Ref	0.255 (0.127, 0.511)	<0.001	0.624 (0.287, 1.356)	0.228	0.149 (0.066, 0.333)	<0.0001	
30–40	Ref	1.029 (0.392, 2.704)	0.952	1.190 (0.533, 2.557)	0.651	0.524 (0.210, 1.309)	0.162	
40–50	Ref	0.498 (0.224, 1.110)	0.087	0.519 (0.245, 1.101)	0.086	0.121 (0.056, 0.261)	<0.0001	
>50	Ref	0.542 (0.231, 1.269)	0.155	0.424 (0.184, 0.977)	0.044	0.114 (0.052, 0.252)	<0.0001	
Gender								0.364
Male	Ref	0.672 (0.399, 1.132)	0.132	0.749 (0.414, 1.354)	0.331	0.169 (0.101, 0.285)	<0.0001	
Female	Ref	0.384 (0.226, 0.652)	<0.001	0.399 (0.207, 0.770)	0.007	0.172 (0.101, 0.293)	<0.0001	
**Handgrip strength (β, 95%CI)**								
Education								0.833
Below high school	Ref	−1.681 (−3.930, 0.568)	0.133	−0.772 (−3.996, 2.452)	0.620	−0.285 (−3.103, 2.533)	0.834	
High school or above	Ref	−0.362 (−2.073, 1.348)	0.661	1.623 (−0.026, 3.272)	0.053	1.835 (0.488, 3.182)	0.011	
PIR								0.610
Not poor	Ref	−0.475 (−2.088, 1.139)	0.543	1.805 (0.141, 3.470)	0.035	1.802 (0.528, 3.076)	0.008	
Poor	Ref	−0.764 (−3.647, 2.120)	0.584	−1.363 (−4.181, 1.455)	0.322	0.415 (−1.812, 2.642)	0.699	
Age								0.059
<60	Ref	−0.847 (−2.110, 0.417)	0.177	1.357 (−0.096, 2.810)	0.066	1.749 (0.643, 2.856)	0.004	
60–80	Ref	−0.830 (−3.406, 1.746)	0.508	−0.190 (−2.866, 2.487)	0.884	−0.334 (−2.661, 1.993)	0.767	
Gender								0.009
Male	Ref	−0.901 (−3.875, 2.073)	0.531	0.694 (−2.569, 3.956)	0.659	3.806 (1.237, 6.375)	0.006	
Female	Ref	0.854 (−0.653, 2.361)	0.248	1.002 (−0.622, 2.627)	0.210	0.637 (−0.915, 2.189)	0.399	

## 4 Discussion

In our study, we conducted a cross-sectional study to elucidate the association between OBS and skeletal muscle mass and strength in 11852 participants of the NHANES cohort. The results showed that OBS was negatively associated with the development of sarcopenia-related traits, with higher OBS being associated with lower values of skeletal muscle mass and strength. In addition, dietary and lifestyle factors were independently associated with skeletal muscle mass and strength.

Numerous studies have investigated the association between major dietary patterns and the risk of skeletal muscle loss disorders. Antioxidants are believed to have a beneficial impact on muscle growth (23). Essential antioxidant nutrients such as vitamins E, vitamins C (24), vitamin B6, vitamins B12, riboflavin (25) are known to play a crucial role in sarcopenia. Sarcopenia was less prevalent among individuals who consumed substantial quantities of magnesium (26), selenium (27), zinc (28), Calcium (29) and copper (30). Adults aged 40 and above who consume higher amounts of dietary fiber have reported improvements in both skeletal muscle mass and strength (31). Santiago et al. demonstrated variations in macronutrient intake (protein, carbohydrates, saturated fatty acids) as well as micronutrient intake (calcium, magnesium, sodium, selenium, and vitamins A, B12, and C) between older adults with and without sarcopenia (32). Nutrients such as vitamins and antioxidants are pivotal in triggering anabolic signaling and protein renewal processes, which are crucial for maintaining muscle function (33–35). The treatment with a derivate, which is commonly found in vitamins and minerals, has been demonstrated to reduce the toxic effects of reactive oxygen species (ROS), improve mitochondrial dysfunction, and enhance muscle strength (36, 37). Vitamin E, vitamin A, zinc, and selenium have been shown to decrease leucine-induced protein breakdown in rats and to increase the anabolic response of muscles to leucine (38). However, a single nutrient may not adequately explain its antioxidant effect on the body. On the other hand, OBS serves as a holistic indicator that better reflects the overall antioxidant status of the body.

Our study also suggests a positive association between lifestyle OBS and skeletal muscle mass. Numerous prior studies have also validated the advantageous effects of physical activity in improving muscle function and/or preventing mobility and somatic limitations (39–41). The Lifestyle Interventions and Independence for Elders (LIFE) demonstrated that a combination of walking and low-intensity resistance training reduced the risk of serious mobility impairments among mobility-impaired older adults (42). This reduction was observed in a dose-dependent manner over a 2-year period when compared to a health education program. Furthermore, the study found that adding at least 48 min of physical activity to weekly routine activities had the most significant benefit. Smoking is recognized to enhance muscle fatigue and interfere with the breakdown of proteins, resulting in a decrease in skeletal muscle mass and function. Additionally, excessive alcohol consumption impairs skeletal muscle protein synthesis, and exposure of muscle to ethanol induces autophagy, contributing to sarcopenia (43). Obesity, especially obesity that reduces muscle strength, increases the risk of falls in older adults, which often requires nutrient intake and increased exercise to improve (44, 45). Therefore, dietary OBS and lifestyle OBS need to be used as a whole to improve skeletal muscle mass and strength.

Furthermore, we observed higher OBS scores among individuals with higher education levels and incomes, indicating a potential positive influence on sarcopenia-related traits. A correlation of positive correlation was observed between adult income and diet quality in Canada (46). Education is often linked with greater nutrition knowledge and the ability to translate that knowledge into healthier dietary habits (47). Compared to individuals with other education levels, adults with a college diploma exhibited higher scores for whole Fruit, total vegetables, whole grains, and calories from solid fats, alcoholic beverages, and added sugars. Higher-income and better-educated people perform better in some health behaviors, such as a greater tendency to have regular medical checkups, eat healthily, exercise, etc. (48, 49). Economic conditions can contribute to the overall well-being, which can then affect behaviors like smoking, physical activity, and food, ultimately leading to a decrease in the occurrence of sarcopenia (50). Interestingly, we found that the positive correlation between OBS and skeletal muscle mass and strength was more significant in people younger than 60 years. Skeletal muscle structure declines progressively with age, and changes in skeletal muscle strength associated with aging begin earlier, i.e., muscle strength begins to decline after age 30 and continues to decline linearly with age (51). By focusing on the prevention of sarcopenia during middle age, individuals can significantly improve their chances of maintaining muscle mass and function, leading to better overall health and quality of life as they age.

Our study used the NHANSE database, a large nationally representative database, to analyze the relationship between OBS and skeletal muscle mass and strength. We also independently investigated the different effects of lifestyle OBS and dietary OBS on skeletal muscle mass and strength. Simultaneously, we conducted subgroup analyses by considering the various features of the population included in the study. However, this study has some limitations. Firstly, it makes a cross-sectional study and cannot accurately determine the causal relationship between OBS and sarcopenia. Additionally, not all variables that affect oxidative stress, like environmental factors, could be included due to database constraints.

## 5 Conclusion

To summarize, a higher OBS indicates that dietary and lifestyle antioxidant exposure surpasses prooxidant exposure and is linked to a reduced risk low skeletal muscle mass and handgrip strength. This discovery implies that following an antioxidative diet and lifestyle may have a possible protecting impact against sarcopenia-related traits, especially on enhancing skeletal muscle mass and handgrip strength. Nevertheless, future research is required to confirm the veracity of our results.

## Data availability statement

The original contributions presented in the study are included in the article/Supplementary material, further inquiries can be directed to the corresponding author.

## Ethics statement

The studies involving humans were approved by Ethical Review Board (IRB) of the National Center for Health Statistics. The studies were conducted in accordance with the local legislation and institutional requirements. Written informed consent for participation was not required from the participants or the participants’ legal guardians/next of kin in accordance with the national legislation and institutional requirements.

## Author contributions

XZ: Data curation, Formal analysis, Software, Writing – original draft. LH: Data curation, Formal analysis, Software, Writing – original draft. KJ: Data curation, Formal analysis, Writing – review & editing. QS: Conceptualization, Supervision, Writing – review & editing. RW: Conceptualization, Methodology, Writing – review & editing.
