# Supplementary material for: Association between oxidative balance score and skeletal muscle mass and strength: NHANES from 2011 to 2018
Source: Front Nutr. 2024 Jun 26;11:1414161. doi: 10.3389/fnut.2024.1414161 (PMC11234853; doi:10.3389/fnut.2024.1414161)
Supplement: Supplementary file 1 [file Data_Sheet_1.docx]

**Table A1**

Weighted univariate logistic regression analysis

**Table A2**

Sensitivity analysis to assess the effects of individual OBS components on the sarcopenia.

Model 3 adjusted for age, race, education, poverty-income ratio, hypertension, diabetes and hyperlipemia.

The specific range for the quantiles is consistent with Table 2.

**Table A3**

Weighted logistic regression analysis between individual dietary components and skeletal muscle mass.

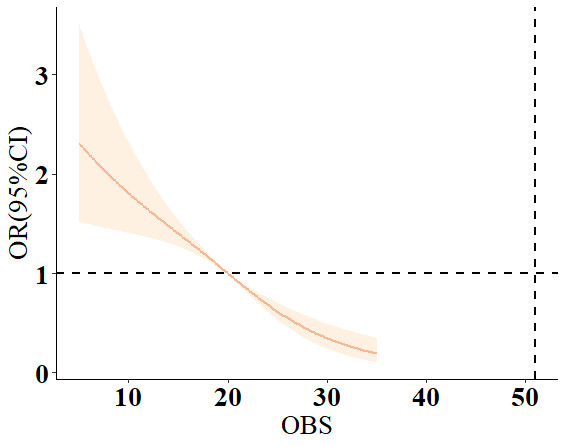


**P for nonlinear=0.0738**

Figure A1. Analysis of restricted cubic spline regression. Legend: Adjusted restricted cubic spline models adjusted for age, race, education, poverty-income ratio, hypertension, diabetes and hyperlipemia.
